# Mutual repression between Pax2 and Snail factors regulates the epithelial/mesenchymal state during intermediate mesoderm differentiation

**DOI:** 10.1242/dev.204848

**Published:** 2025-08-15

**Authors:** Juan M. Fons, Oscar H. Ocaña, M. Angela Nieto

**Affiliations:** ^1^ Instituto de Neurociencias (CSIC-UMH), Avda. Ramón y Cajal s/n, Sant Joan d'Alacant, Spain

**Keywords:** Intermediate mesoderm, Pax2, Snail1, Bi-stability domain, EMT, MET, Chick

## Abstract

The pronephros is the first renal structure in the embryo, arising by mesenchymal-to-epithelial transition of the intermediate mesoderm, where Pax2 induces epithelialization and differentiation of this mesenchyme. Here, we show in chick embryos that Snail1 is sufficient to keep the intermediate mesoderm in an undifferentiated state by directly repressing *Pax2* transcription. Reciprocally, Pax2 is sufficient and necessary to induce mesenchymal-to-epithelial transition by directly repressing *Snail1* expression. We also show that BMP7 acts as an epithelialization signal by downregulating *Snail1* and upregulating *Pax2* expression. This, together with the Snail1/Pax2 reciprocal repression, establishes a regulatory loop within the bi-stability domain, a dynamic region of the anteroposterior axis where opposing retinoic acid/fibroblast growth factor gradients converge, and which has been found to regulate differentiation of the neural tube and somites. In conclusion, we show that the antagonism between Snail1 and Pax2 determines the epithelial/mesenchymal state during the differentiation of the intermediate mesoderm and propose that the bi-stability domain extends to the intermediate mesoderm, synchronizing the differentiation of all tissues aligned along the mediolateral embryonic axis.

## Teaser

**Summary:** The antagonism between Snail and Pax2 factors in the bi-stability domain tightly regulates the timing of mesodermal epithelialization in chick embryos.

## INTRODUCTION

During embryonic development, most of the tissues are derived from the sequential activation of epithelial-mesenchymal (EMT) and mesenchymal-epithelial (MET) transitions (Thiery et al., 2009). A prototypic example is the development of the sequential renal structures pronephros and metanephros. The pronephros is the first epithelial structure of the urogenital system that arises from the intermediate mesoderm (IM) after MET. It encompasses the nephric duct and nephric cord running along both sides of the embryo adjacent to the somites. It elongates posteriorly until it reaches the metanephric mesenchyme where the adult kidney will develop (Dressler, 2009; James and Schultheiss, 2003).

Elongation of the pronephros along the anterior-posterior (AP) axis is in synchrony with a wave of differentiation in the same axis of the neural tube and the somites (Aulehla and Pourquié, 2010; Del Corral and Storey, 2004). The mechanism involves a fibroblast growth factor (FGF)/retinoic acid (RA) counter-gradient (Aulehla and Pourquié, 2010; Del Corral et al., 2003) that controls the initiation of differentiation along the AP axis at a region known as the transition zone. Mathematical modelling has shown that the mutual inhibition of FGF and RA signalling generates a bi-stability domain (BD) in the transition zone whereby cells can be in one of two steady states. Cells with higher FGF signalling are maintained mesenchymal and undifferentiated, and those with higher RA signalling switch to epithelial differentiation leading to sharp developmental thresholds (Goldbeter et al., 2007).

During the development of the renal system, Snail genes are expressed in the IM before pronephros epithelialization. They are also expressed later in the metanephric mesenchyme prior to the MET that leads to the formation of the renal vesicle (Boutet et al., 2006). In both cases, epithelialization concurs with the repression of Snail factors, which are maintained in a silent state in the adult, although they are reactivated in pathological conditions such as fibrosis (Grande et al., 2015; Lovisa et al., 2015). By contrast, *Pax2* is an essential promoter of MET and is required for the specification of the renal epithelial lineage (Bouchard et al., 2002; Dressler et al., 1993; Torres et al., 1995). It is expressed in the IM just prior to MET and continues to be expressed in the epithelial derivatives of the pronephros, mesonephros and metanephros (Bouchard et al., 2000, 2002; Dressler et al., 1990; Dressler and Douglass, 1992; Narlis et al., 2007). Another important molecule that promotes epithelialization and MET during renal development is BMP7, a member of the TGFβ superfamily (Massagué, 2012) expressed in the pronephros, mesonephros and metanephros (Lyons et al., 1995; Vukicevic et al., 1996). In agreement with this, *Bmp7* null mutant mice display renal hypoplasia with a reduction of the epithelial compartment (Dudley et al., 1995; Luo et al., 1995). Furthermore, BMP7 treatment prevents and reverses acute renal fibrosis, restructuring the epithelial tubules of the kidney, inducing MET by antagonizing TGFβ signalling (Sato et al., 2003; Zavadil et al., 2004; Zeisberg et al., 2003, 2005), which induces *Snail1* expression (Peinado et al., 2003).

Here, we have examined the relationship between the EMT inducer Snail1 (Snai1) and the MET inducers Pax2 and BMP7 in the chick IM, as this is an excellent model in which to study epithelial plasticity in the embryo. We found that reciprocal repression between Snail1 and Pax2 controls the timing of differentiation. This negative regulatory loop is triggered by BMP7, which simultaneously represses *Snail1* and activates *Pax2* expression. This loop is activated in the BD, where differentiation of the neural tube and the paraxial mesoderm also occurs, indicating that it is integrated in the synchronization of differentiation processes along the mediolateral axis at a given AP level.

## RESULTS AND DISCUSSION

### *Snail1* and *Pax2* are expressed in mutually exclusive domains in the developing mesoderm

**Fig. 1.**
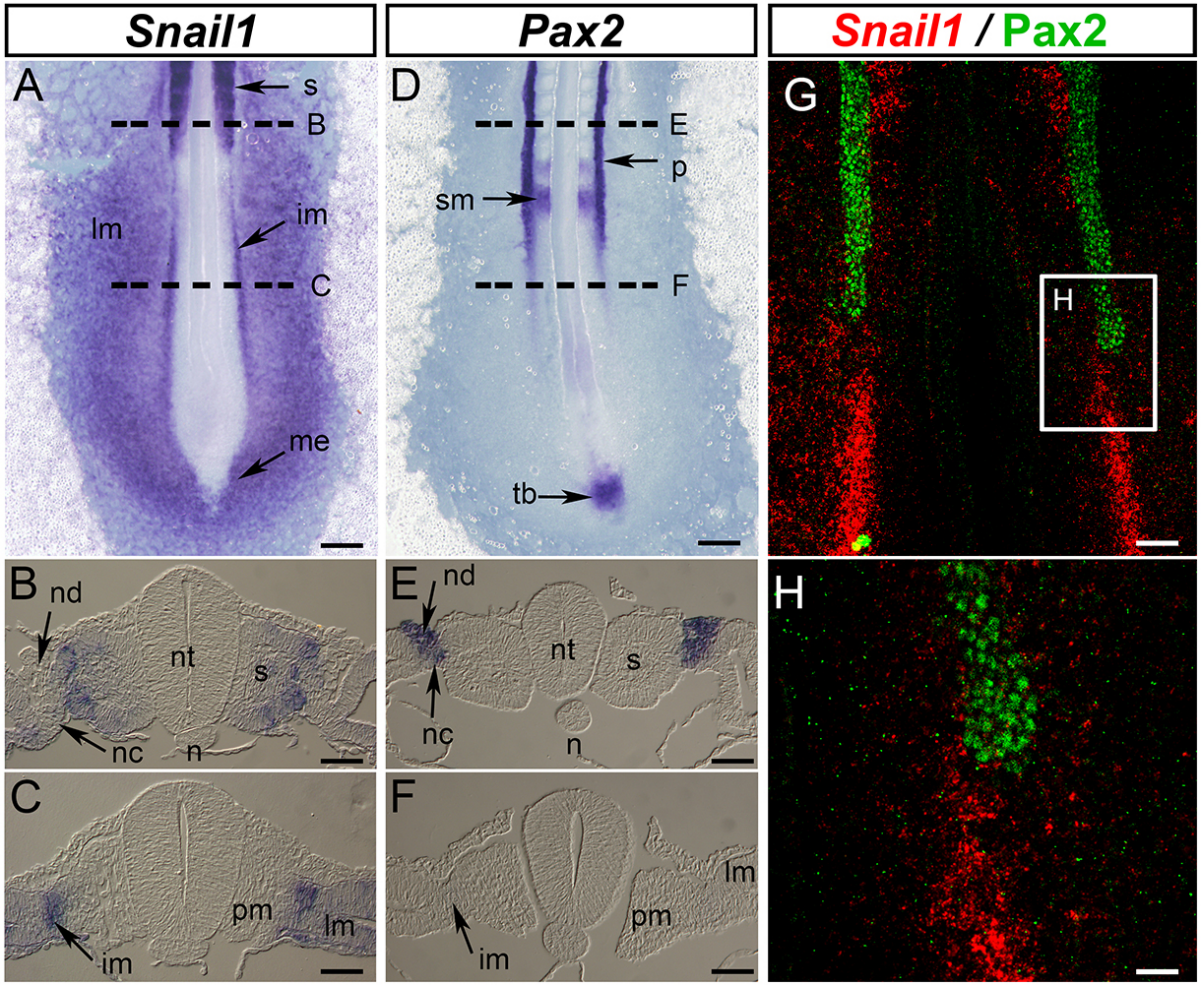
***Snail1* and *Pax2* are expressed in a mutually exclusive pattern in the developing embryo.** (A-F) Dorsal view of chicken embryos (HH11) and transversal sections taken at somite +2 (B,E). The images in C and F were taken 500 µm posteriorly to the last somite (+1) (indicated by the dashed lines). (A-C) *Snail1* is expressed in the ingressing mesendoderm (me), undifferentiated intermediate mesoderm (im), lateral plate mesoderm (lm) and somites (s) (*n*=10). (D-F) *Pax2* is expressed in the pronephros (p), somitomeres (sm) and tail bud (tb) (*n*=10). (G) 3D reconstruction of *in situ* hybridization for *Snail1* (red) and immunofluorescence for Pax2 (green) at HH11 (*n*=3). (H) Higher magnification of the boxes area in G. n, notochord; nc, nephric cord; nd, nephric duct; nt, neural tube; pm, paraxial mesoderm. Scale bars: 150 µm in A,D; 50 µm in B,C,E,F; 75 µm in G; 30 µm in H.

Given that the silencing of *Snail1* expression correlates with the epithelialization of the metanephros (Boutet et al., 2006) and that *Pax2* is required for the epithelialization of pro- and metanephros (Bouchard et al., 2002; Narlis et al., 2007), we decided to compare the expression of both factors at early stages of mesoderm development in the chick embryo (Fig. 1). We found that *Snail1* is expressed in mesenchymal tissues, including the undifferentiated intermediate and lateral mesoderm (Fig. 1A-C) but absent from the nephric duct and cord of the pronephros (Fig. 1B). By contrast, *Pax2* is expressed in the epithelial structures of the pronephros but not in the mesenchymal IM (Fig. 1D-F). We performed fluorescence *in situ* hybridization (ISH) for *Snail1* (red) and immunofluorescence for Pax2 (green) and confirmed the absence of *Snail1/Pax2* co-expression (Fig. 1G,H). We also found that the complementary expression is observed even before the formation of the nephric duct (Fig. S1), indicating that *Snail1* and *Pax2* expression domains are mutually exclusive in the developing embryo.

### Snail1 prevents epithelialization of the IM by directly repressing *Pax2* transcription

**Fig. 2.**
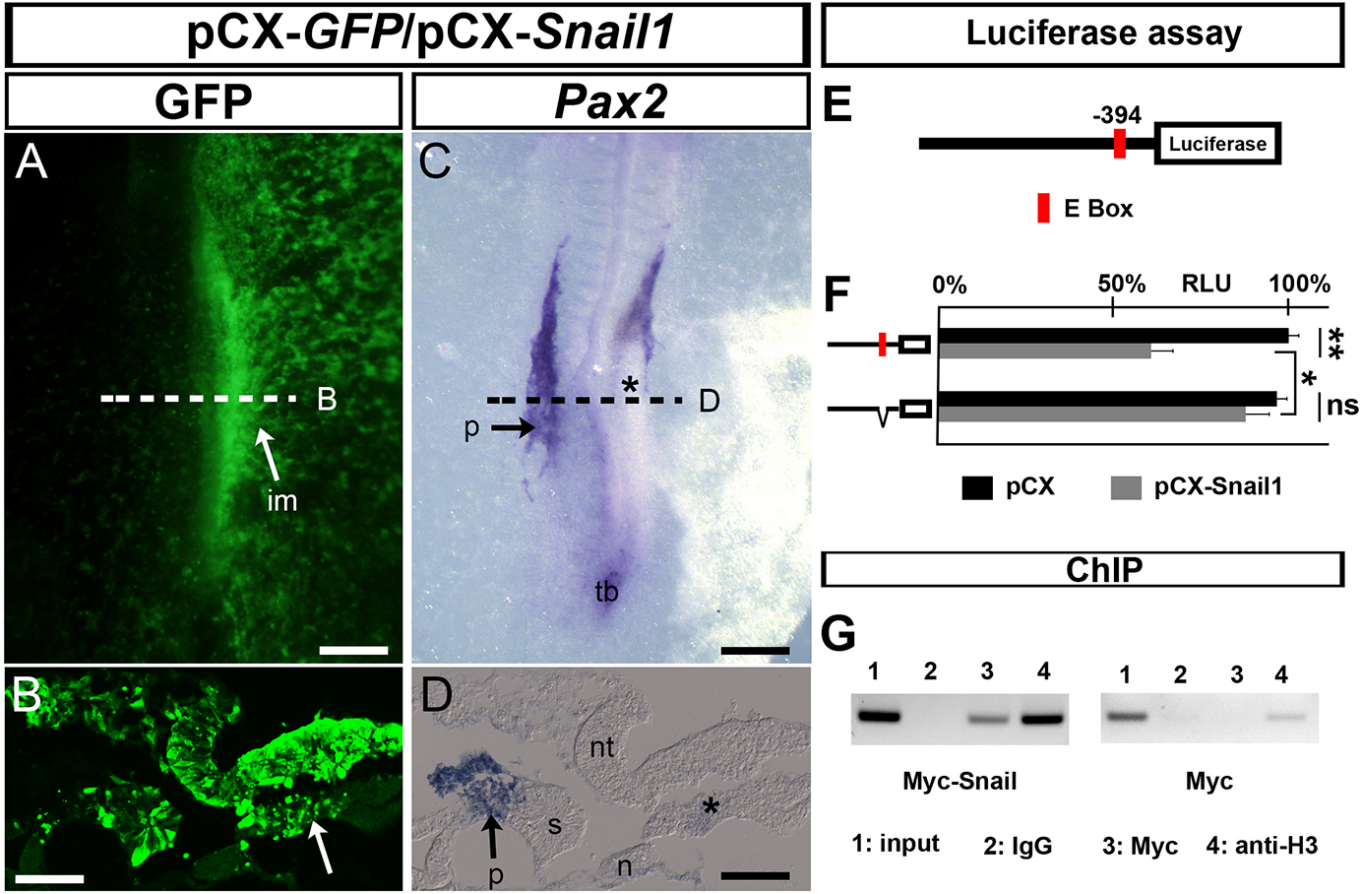
**Snail1 is a direct repressor of *Pax2* transcription.** (A-D) Chick embryos co-electroporated on the right side with a vector encoding either GFP (pCX-*GFP*) or the *Snail1* coding region (pCX-*Snail1*). Dorsal view of HH11 chick embryo and sections showing GFP immunofluorescence (A,B) and *Pax2* expression. Sections in B and D were taken at somite +2 (indicated by the dashed lines). Snail1 ectopic expression represses endogenous *Pax2* expression (C,D) in the intermediate mesoderm (im; A,B, white arrows; C,D, black asterisks) (*n*=9/9). (E) Scheme of the *Pax2* promoter fragment (1.8 kb upstream of the TSS) with the Snail1-binding site (E box) shown in red. (F) Luciferase assay in HEK293T cells transfected with a vector containing a fragment of the *Pax2* promoter bearing the wild-type (red) or a mutated version of the E box plus either pCX (black bars) or pCX-*Snail1* (grey bars) vectors. Snail1 decreases *Pax2* promoter activity, and the mutated E box significantly restores activity (*n*=3, average representation). *P<0.01, **P<0.001 (*t*-test; two tails, unequal variance). ns, not significant. (G) ChIP assay in chick embryos electroporated with a Myc-tagged Snail1-coding plasmid (Myc-Snail1) or an empty Myc plasmid (Myc) (*n*=3, representative experiment shown). (1) Input; (2) IgG antibody (IgG), negative control; (3) Myc antibody (anti-Myc); (4) Histone3 antibody (anti-H3), positive control. There is amplification of DNA fragments containing the E box in the presence of Myc-Snail1. n, notochord; nt, neural tube; p, pronephros; s, somites; tb, tail bud. Scale bars: 150 µm in A,C; 50 µm in B,D

The expression pattern of *Snail1* and *Pax2* and their described roles in EMT/MET regulation already suggested a possible genetic interaction. To investigate this, we first performed gain-of-function experiments by co-electroporating plasmids coding for Snail1 and GFP and analysed *Pax2* expression (Fig. 2A-D). The regions of the IM with *Snail1* ectopic expression (Fig. 2A,B, green) showed a downregulation of *Pax2* expression compared with the control electroporation and the contralateral side (Fig. 2C,D arrows and asterisk; Figs S2A-D and S3). When only the ectoderm was targeted, *Pax2* expression was not downregulated in the IM (Fig. S3B,E,H,K,N,Q, arrowheads) in contrast to when the IM was electroporated (Fig. S3C,F,I,L,O,R), suggestive of a cell-autonomous effect. Analysis of the *Pax2* promoter showed an E box compatible with Snail1 binding (Cano et al., 2000) (Fig. 2E). Luciferase assays in the *Pax2-*positive human renal embryonic epithelial HEK293T cells (Tamimi et al., 2008) showed that Snail1 can repress *Pax2* promoter activity (Fig. 2F; Fig. S4A-C). Furthermore, mutation of the E box into a sequence to which Snail1 does not bind (Batlle et al., 2000) restored promoter activity (Fig. 2F). Chromatin immunoprecipitation (ChIP) assays from chick embryo tissues obtained after electroporation of a plasmid encoding a Myc-tagged Snail1 version (Acloque et al., 2011) confirmed enrichment of Snail1 binding to the *Pax2* promoter (Fig. 2G), indicating that Snail1 behaves as a direct repressor of *Pax2* expression *in vivo*. Thus, *Snail1* expression can maintain the IM in an undifferentiated state by preventing *Pax2* expression, thereby avoiding the premature epithelialization of the pronephros. This is in agreement with the finding that the IM fails to become epithelial and remains mesenchymal in *Pax2* mutant mice (Torres et al., 1995). As *Snail1* endogenous silencing in the metanephric mesenchyme correlates with MET and renal differentiation (Boutet et al., 2006), and differentiation relies on *Pax2* upregulation (Narlis et al., 2007), it is likely that *Snail1* also prevents premature metanephros differentiation by repressing *Pax2* expression, in addition to its previously described role in repressing *Hnf1b* and cadherin 16 (*Cdh16*) transcription (Boutet et al., 2006).

We then wondered whether *Snail1* silencing was sufficient to upregulate *Pax2* expression in the IM. Electroporation of an interference RNA against *Snail1* repressed its expression in the electroporated regions (Fig. S5A-D), but *Pax2* was not induced (Fig. S5E-H). This indicates that *Snail1* repression is not sufficient to activate *Pax2* transcription, in line with the role of Snail1 as an epithelial repressor without instructing a particular cell fate, as previously shown for *Snail2* (Snai2) at gastrulation stages during mesoderm formation (Acloque et al., 2011).

### A Snail1/Pax2 reciprocal inhibitory loop regulates the differentiation of the IM

**Fig. 3.**
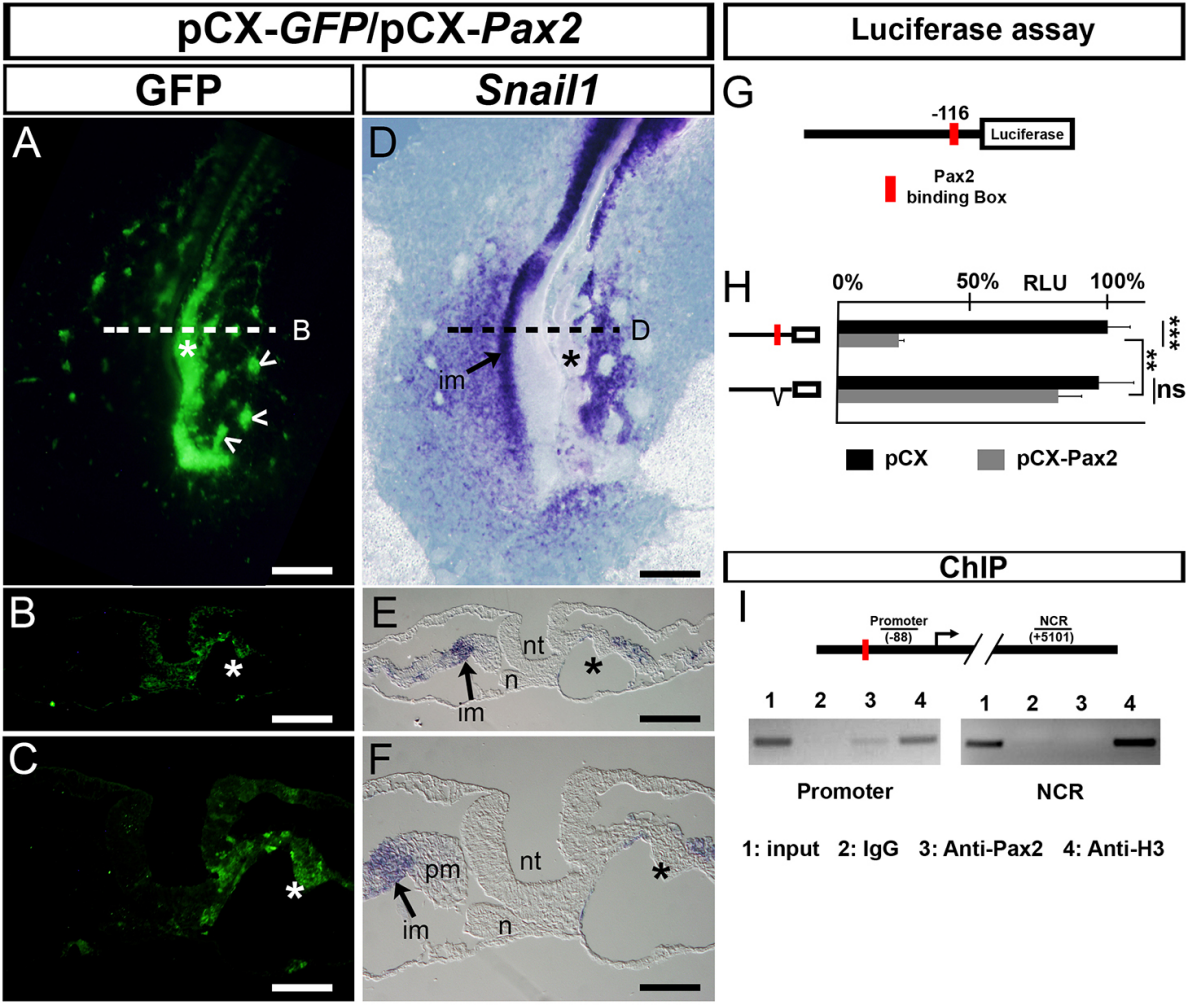
**Pax2 is a direct repressor of *Snail1* transcription.** (A-F) Electroporation on the right side of a chick embryo with pCX-*GFP* plus pCX-*Pax2* vectors showing immunofluorescence for GFP (A-C) and ISH for *Snail1* (D-F). (A,D) Dorsal views of HH11 chick embryo. Arrowheads indicate cellular aggregates. (B,C,E,F) Sections taken at the level of the dashed lines [500 µm posterior to the latest somite formed (+1)]. *Pax2* ectopic expression (GFP) represses *Snail1* expression in the intermediate mesoderm (asterisks) (*n*=18/18); this can be more clearly observed in the higher magnification images (C,F). (G) Scheme of the *Snail1* promoter fragment (0.15 kb upstream of the TSS) with the Pax2-binding box shown in red. (H) Pax2 decreases *Snail1* promoter activity, and deletion of the box restores it (*n*=3, average representation). **P<0.001, ***P<0.0001 (*t*-test; two tails, unequal variance). ns, not significant. (I) ChIP assay. Scheme showing the PCR fragments analysed, one in the promoter region and another one from a non-coding region (NCR). (1) Input; (2) IgG antibody (IgG), negative control; (3) Pax2 antibody (anti-Pax2); (4) Histone3 antibody (anti-H3), positive control. (*n*=3, representative experiment shown). im, intermediate mesoderm; n, notochord; nt, neural tube; pm, paraxial mesoderm. Scale bars: 200 µm in A,D; 100 µm in B,E; 50 µm in C,F.

As Pax2 is required for MET and renal differentiation (Bouchard et al., 2002; Rothenpieler and Dressler, 1993; Torres et al., 1995) and *Snail1* silencing is essential for pronephros differentiation (Fig. 2), we wondered whether Pax2 promotes MET in the IM at least in part by repressing *Snail1* transcription. We performed gain-of-function experiments for Pax2 as described above for Snail1 (Fig. 3A-F; Fig. S2E-H). The areas of *Pax2* ectopic expression (Fig. 3A-C, green) were devoid of *Snail1* transcripts (Fig. 3D-F; asterisks indicate the effect on the IM). Interestingly, the electroporated mesoderm showed cellular aggregates compatible with a MET process (Fig. 3A, arrowheads), confirmed by the re-expression of E-cadherin (cadherin 1) in the electroporated area (Fig. S6). Interestingly, these aggregates are reminiscent of the multicellular rosettes that guide MET, leading to the emergence of epithelial structures in other mesodermal populations, such as the notochord and lateral plate mesoderm (Abboud Asleh et al., 2023; Gredler and Zallen, 2023). Pax2 activation was previously shown to induce ectopic nephric structures (Bouchard et al., 2002), but these results also show that the sole activation of Pax2 expression in Snail1-positive intermediate and lateral mesoderm is sufficient to induce epithelialization. Thus, we examined whether Pax2 could also act as a *Snail1* repressor. A luciferase assay in primary cultures of chick embryonic fibroblasts showed that the co-transfection of a Pax2-encoding vector with serial DNA fragments upstream the transcription start site (TSS) repressed *Snail1* promoter activity (Fig. S4D-G). We identified a Pax2-binding site 116 bp upstream of the *Snail1* TSS (JASPAR database) within the minimal fragment of 150 bp of *Snail1* promoter. Pax2 was sufficient to repress *Snail1* minimal promoter activity in a dose-dependent manner (Fig. 3G,H; Fig. S4D-F). Deletion of the Pax2-binding site impaired this repression (Fig. 3H). We next performed ChIP analysis in chick embryo tissues and confirmed that Pax2 can bind to the *Snail1* promoter *in vivo* (Fig. 3I). Then, we examined whether Pax2 was not only sufficient but also required for *Snail1* downregulation, which we confirmed after finding that *Pax2* silencing by iRNA electroporation led to *Snail1* maintenance, in contrast with the downregulation observed in the contralateral control side (Fig. S7).

Altogether, these data show that, like Snail1 repressing *Pax2* transcription, Pax2 represses *Snail1* transcription by direct binding to its promoter. Thus, Snail1 and Pax2 establish a reciprocal negative loop, a mechanism widely used during development when fate decisions involve binary choices. This is observed in the subdivision of embryonic territories, including the decision to become trophectoderm or inner cell mass (Cdx2/Oct4) or ectodermal versus mesendodermal (SoxB1/Snail) (Acloque et al., 2011, 2012; Niwa et al., 2005). This reciprocal repression between Snail1 and Pax2 is also likely to occur during metanephros development and may also have a correlate in pathological conditions. *Pax2* reactivation occurs in fibrotic or injured kidneys (Hou et al., 2018; Imgrund et al., 1999), and this is thought to be an attempt to recover the normal renal epithelial architecture either through MET of the renal mesenchyme or by increasing proliferation of the remaining epithelial cells (Lindoso et al., 2009). We propose that this role of *Pax2* reactivation may be achieved by counteracting the activation of *Snail1* in renal epithelial cells upon injury (Grande et al., 2015).

### BMP7 controls pronephros differentiation by repressing *Snail1* and inducing *Pax2* expression in the BD

**Fig. 4.**
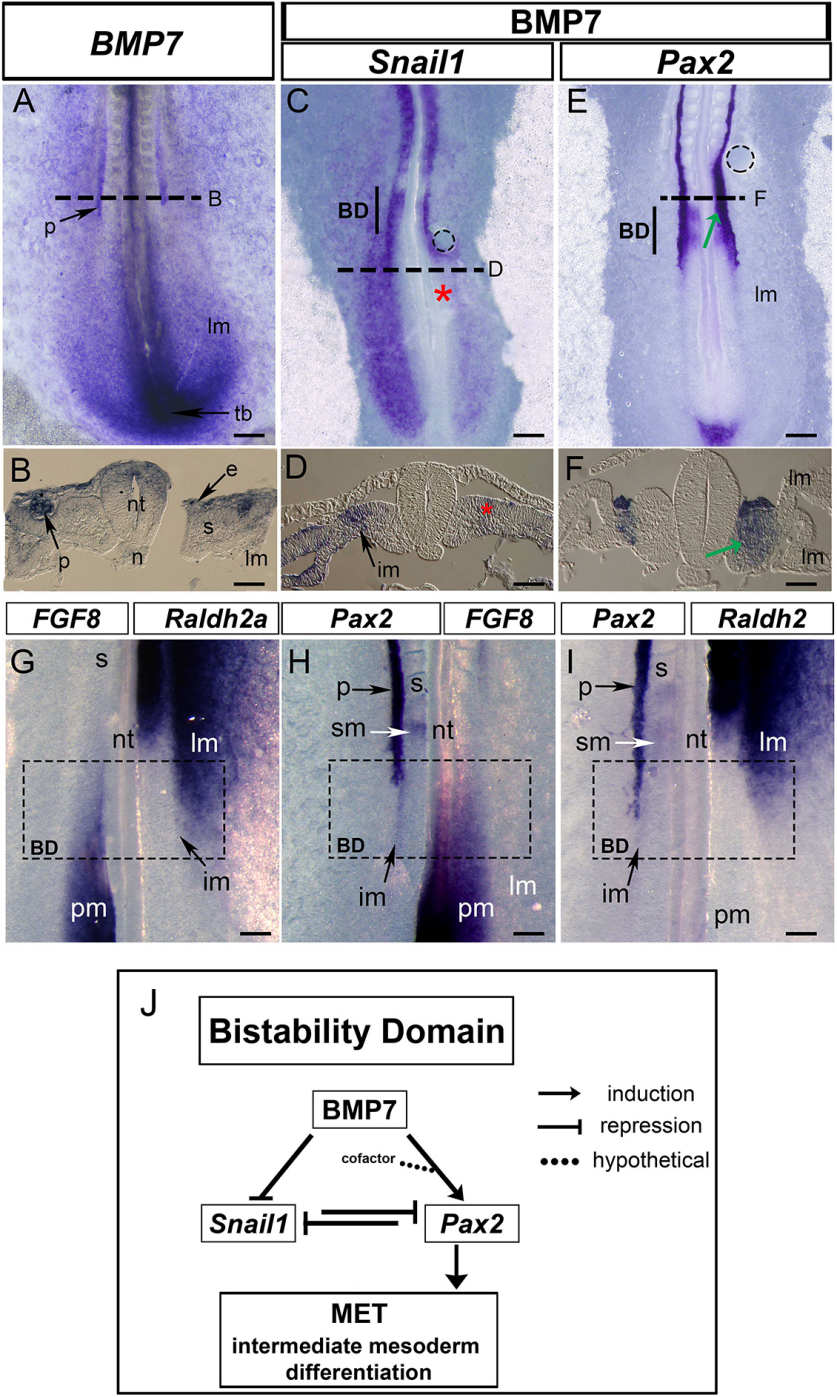
**BMP7 represses *Snail1* transcription and induces *Pax2* expression in the bi-stability domain.** (A,B) ISH for *BMP7* and the respective transverse section (taken at somite +2, HH11 chick embryo; indicated by the dashed line). *BMP7* is expressed in the ectoderm (e), dorsal neural tube (nt), pronephros (p) and tail bud (tb). (C-F) HH11 chick embryos bearing a bead (dashed circle) previously soaked in BMP7 (50 µg/ml) and subjected to ISH for *Snail1* (C,D) or *Pax2* (E,F). Sections taken 500 µm posterior to the latest somite formed (D) or at somite +2 (F) (indicated by the dashed lines). BMP7 represses *Snail1* in the mesoderm (C,D, asterisks; *n*=3/5) and induces *Pax2* expression in the bi-stability domain (E,F, arrows; *n*=3/5). (G-I) Sagittal halves of HH11 chick embryos subjected to an ISH for different gene pairs. (G) *Fgf8* (left) and *Raldh2* (right), (H) *Pax2* (left) and *FGF8* (right), and (I) *Pax2* (left) and *Raldh2* (right). The intersection of paired genes expression defines the bi-stability domain (BD), at the level of pronephros differentiation front. (J) Model for pronephros differentiation. BMP7 directs the flow of information by repressing *Snail1* and inducing *Pax2* to promote renal differentiation through MET in the intermediate mesoderm within the BD. A mediolateral gradient of BMP4 from the lateral mesoderm together with a gradient of noggin from the notochord restricts the induction of *Pax2* by BMP7 to the intermediate mesoderm. The anteroposterior gradient of retinoic acid and FGF8 restricts *Pax2* induction to the bi-stability domain. im, intermediate mesoderm; lm, lateral mesoderm; n, notochord; pm, paraxial mesoderm; s, somites; sm, somitomeres. Scale bars: 150 µm in A,C,E; 50 µm in B,D,F; 100 µm in G-I.

Next, we investigated signalling pathways that could be regulating these genes. We focused on BMP members as they induce pronephros differentiation (Bracken et al., 2008; James and Schultheiss, 2005). Specifically, we tested BMP7, as it is also required for MET and differentiation of the metanephros (Dudley et al., 1995; Luo et al., 1995; Vukicevic et al., 1996), and it is expressed in the pronephros, among other tissues (Fig. 4A,B). We challenged chick embryos by administering BMP7 and assessed its impact on *Snail1* and *Pax2* expression.

BMP7 behaved as a very strong *Snail1* transcriptional repressor in the mesoderm all along the AP axis (Fig. 4C,D, asterisks; Figs S8A-H and S9). In addition, it induced *Pax2* transcription in the paraxial/somitic mesoderm (Fig. 4E,F, arrows; see also Fig. S8I-P), and repressed the somite marker *Paraxis* (*Tcf15*) (Fig. S10). This shows that ectopic BMP7 disrupts the mediolateral gradient of BMP signalling (Greenfeld et al., 2021; James and Schultheiss, 2005) and transforms paraxial mesoderm into IM, expressing *Pax2* now adjacent to the neural tube. It is worth noting that BMP7 did not induce *Pax2* in posterior regions, where it still repressed *Snail1*. Therefore, the repression of *Snail1* by BMP7 in these territories is *Pax2* independent (Fig. S11). In addition, *Snail1* loss was not sufficient to induce *Pax2* expression (Fig. S5) in territories described to express BMP7, including the posterior IM (Fig. 4E,F; Fig. S8I-P). Thus, BMP7 does not induce *Pax2* solely through *Snail1* repression, but concomitant with it. In summary, BMP7 induces epithelialization and differentiation of the IM through two independent mechanisms: (1) repressing the EMT inducer *Snail1*, and (2) inducing the MET inducer *Pax2*. These data and expression studies suggest that BMP7 may also control metanephros epithelialization and differentiation through the same mechanism, and that the mechanism behind the attenuation of renal fibrosis by BMP7 (Zeisberg et al., 2003, 2005) is likely mediated by the repression of *Snail1* and the induction of *Pax2*.

The ability of BMP7 to activate *Pax2* expression in the paraxial mesoderm occurred in a specific region of the anteroposterior axis of the embryo (Fig. 4E,F; Fig. S8I-P), which looked reminiscent of the BD, defined as the intersection of expression domains for *Raldh2* (*Aldh1a2*) and *FGF8*, two morphogens responsible for patterning the anteroposterior axis (Del Corral and Storey, 2004), and that delimits the region where cells switch from an undifferentiated to differentiated state (Goldbeter et al., 2007). We examined the expression of the two morphogens in relation to that of *Pax2* in halves of chick embryos (Fig. 4G-I) and found that the differentiation front of the IM, assessed by *Pax2* expression, resides within the BD (Fig. 4H,I). The initial location of the BMP7 bead in the experiment shown in Fig. 4E was within the BD where *Pax2* is first induced in the paraxial mesoderm. As the BD moved caudally, *Pax2* was induced dynamically along the anteroposterior axis (Fig. 4E,F; Fig. S8I-P). Moreover, we did not observe *Pax2* induction in territories that are naturally incompetent to express it, such as the lateral mesoderm, or posterior to the BD (Fig. 4E,F). This is compatible with the described competence of the cells to respond to BMP according to the embryonic anteroposterior level, synchronizing tissue differentiation in the different axes (Tucker et al., 2008). As such, the response is within the transition zone, and restricted to the BD (Fig. 4E,F, arrows and Fig. S8I-P). As mentioned, the BD is defined as the area of convergence of two opposing anteroposterior gradient of retinoic acid and a posteroanterior gradient of FGF/Wnt within the anterior one-third of the transition zone (Aulehla and Pourquié, 2010; Del Corral and Storey, 2004; Goldbeter et al., 2007; Olivera-Martinez and Storey, 2007), where neurogenesis (Del Corral and Storey, 2004) and somitogenesis (Aulehla and Pourquié, 2010) occur. Differentiation at the BD is perfectly compatible with the region where binary choices occur (Goldbeter et al., 2007), and particularly with the establishment of reciprocal negative loops as we describe here for Snail1 and Pax2.

Interestingly, retinoic acid induces pronephros differentiation (Cartry et al., 2006; Wingert et al., 2007) and represses *Snail1* during somite epithelialization (Morales et al., 2007). In addition, *Snail1* is downstream of the Wnt/FGF signalling pathway during somitogenesis, and it needs to be repressed for epithelialization to occur (Dale et al., 2006). Our results show that the BD not only regulates neurogenesis and somitogenesis but also IM lineage commitment. We propose a model for pronephros differentiation (Fig. 4J) in which BMP7 represses *Snail1* expression and induces *Pax2* transcription in the IM, where the appropriate BMP levels exist. The levels are regulated by two opposing medio-lateral gradients of high noggin from the notochord/dorsal neural tube and high BMP4 from the lateral mesoderm, which pattern the embryo along the medio-lateral axis (James and Schultheiss, 2005). *Snail1* maintains the undifferentiated state of the IM in the posterior part of the embryo, and at the BD domain Snail1 and Pax2 stablish a negative reciprocal loop that leads to epithelialization. Thus, we extend to the IM the bi-stability concept, leading to differentiation in the anteroposterior axis, synchronizing the different cell populations along the mediolateral axis of the embryo.

## MATERIALS AND METHODS

### Chick embryonic fibroblast isolation

Muscle fragments from HH35 chick embryos were dissected in PBS at 37°C and disaggregated with trypsin for 5 min at room temperature (RT). The homogenates were centrifuged at 1100 rpm (282 ***g***) for 6 min and the pellets were suspended in F-12/HAM and seeded on p10.

### Cell culture

HEK293T cells were cultured in DMEM (Sigma-Aldrich, D6429) with 10% fetal calf serum (Invitrogen, 10106-169), 1% penicillin/streptomycin (Sigma-Aldrich, P4333) and 1% fungizone (Sigma-Aldrich, A2942). Chick embryonic fibroblasts (CEFs) were cultured in F-12/HAM (Sigma-Aldrich, N6658) with 10% fetal calf serum, 1% penicillin/streptomycin plus fungizone. Both primary and established cell lines were tested negative for contamination with *Mycoplasma*.

### Cell transient transfection and luciferase assays

HEK293T cells and CEFs were cultured at 10^5^ cells/well in a 12 multiwell plate for 24 h prior to transfection. For luciferase assays, a reaction mix in a final volume of 50 µl (for HEK293T) or 100 µl (for CEFs) in Opti-MEM (Invitrogen, 51985) was made, respectively, containing 400 ng of pGL3basic-*Pax2* promoter or 800 ng of pGL3b-*Snail1* promoter vectors and 40 ng of CMV-*Renilla* plasmid as endogenous control. To this mix, 50, 100 or 200 ng of pCX-*Snail1* or 20, 50 or 100 ng of pCX-*Pax2* vectors were added to HEK293T cells or CEFs, respectively. FuGENE HD Transfection Reagent (FuGENE, E2312) was used for HEK293T cells and Lipofectamine Transfection Reagent (ThermoFisher Scientific, 18324-012) for CEFs. After adjusting the culture medium to 500 µl, the reaction mix for HEK293T was added and cells were assayed for luciferase activity after 48 h. For CEFs, the reaction mix was added to 200 µl of opti-MEM for 5 h and the medium was replaced with DMEM afterwards. After the transfection, cells were washed twice in PBS and assayed following the manufacturer's instructions (Promega, Dual-Luciferase Reporter Assay System, TM040).

### Embryo electroporation

Electroporations were performed as described by Acloque et al. (2011). For gain-of-function experiments, embryos at Hamburger–Hamilton stage (HH) 3-4 were electroporated with 2 µg/µl of pCX-*Pax2* or pCX-*Snail1* and 0.5 µg/µl of pCX-*EGFP* and cultured until HH11 using the easy culture system.

For loss-of-function experiments, embryos at HH4 were electroporated with siRNA at 1 µM and 0.5 µg/µl of pCX-EGFP. The sequence of the siRNA against *Snail1* was 5′-CCUUUCCCGUGCAGAUACAUGUAUU-3′ and the siRNA against *Pax2* was 5′-ACCUGACGUGGUGAGACAAAGGAUA-3′.

### ISH

We used a protocol previously described (Nieto et al., 1996) for non-radioactive ISH. For fluorescence ISH in combination with immunofluorescence (anti-Pax2 at 1/200; Abcam, Ab23799), we followed the method described by Acloque et al. (2008).

### Cytokine treatment

Heparin acrylic beads (Sigma-Aldrich, H5263) were embedded in a BMP7 solution (recombinant human BMP7; R&D Systems, 354-BP) in PBS. Beads were added to embryos at stage HH9 for 8 h before fixing the embryos.

### Gene cloning and mutagenesis

We used three chick embryos at HH11 for DNA genomic extraction with chloroform. A 2 kb DNA fragment upstream of the TSS of the *Snail1* promoter (ENSGAL00000008018) was cloned into a pGL3 basic plasmid (Promega) using PWO polymerase (Roche, 11644947001) and NheI and HindIII (New England Biolabs) target sequences flanking the forward (5′-AAAAAAGCTAGCACCGGGTCTACTTGAATTTTG-3′) and reverse (5′-AAAAAAAAGCTTCGTACTCGCCCAGCGCCACCG-3′) primers, respectively. To generate subsequent deletions in the *Snail1* promoter, we used the construct mentioned above as a template and the same restriction enzymes target sequences flanking the primers. The primers used were: 1.2 kb fragment forward primer (5′-AAAAAAGCTAGCGAATTACGGCAATTG-3′), 0.6 kb fragment forward primer (5′-AAAAAAGCTAGCCCCGCTTCAGTGGG G-3′) and 0.15 kb fragment forward primer (5′-AAAAAAGCTAGCTAGTCTGCCCGCCCCGG-3′) with the above-described reverse primer.

For the deletion of the Pax2 binding site located at positions −116/−109 from the TSS in the *Snail1* promoter, we used self-complementary primers (forward 5′-CGTCCCATTGGCTCCGGGGGCGGCCCTGCACCGCCCTC-3′, reverse 5′-GAGGGCGGTGCAGGGCCGCCCCCGGAGCCAATGGGACG-3′) without the Pax2 target sequence (5′-GGGCATGG-3′) and carried out whole plasmid amplification. The PCR product was digested with DnpI (New England Biolabs) for 5 h and transfected into *Escherichia coli* DH5α strain. To subclone the *Pax2* promoter (NM_204793.1), we used a bacterial artificial chromosome (BAC; CH261-43k16) containing the *Pax2* locus. Since the chick *Pax2* promoter sequence was not annotated, we identified a conserved region at *Pax2* locus of *Gallus gallus* using the ECR-Browser (http://ecrbrowser.dcode.org) (galGal3 chrUn_random:58143187-58143360), compared this sequence with the chick whole genome by Blastn, (http://blast.ncbi.nlm.nih.gov/Blast.cgi; Traces-WGS) and we found a contig with a fraction of the chick *Pax2* promoter (Nw_001478025.1). PvuII (New England Biolabs) and NcoI digestion, respectively, inside and at the TSS allowed us to obtain a BAC fragment containing the *Pax2* promoter. This was confirmed by Southern blot performed with two α-P32-dCTP-labelled probes, one at Nw_001478025.1 (forward primer 5′-AAAGAGACGGAGAAGTGTATTTCG-3′, reverse primer 5′-ACGTTTGAGAAAAACAAAGGAACT-3′) and another one at 5′UTR (forward primer 5′-ATTGCTTTGCTTTGGTTTGTTATT-3′, reverse primer 5′-GAGGCAAAGGAAAGGGAAGA-3′). After isolating the identified band, it was ligated into pGL3 basic plasmid, and transfected into *E. coli* DH5α that were hybridized with the same probe for confirmation, obtaining a 1.7 kb DNA fragment upstream of the TSS of the chick *Pax2* promoter. The cloned sequence is given in supplementary Materials and Methods and has now been annotated in Ensembl.

For the mutagenesis of the Snail1-binding site (E-box) in the *Pax2* promoter, we mutated the E-box sequence (5′-GCAGCTG-3′ converted to 5′-TTAGCTA-3′) by PCR with the following primers: forward 5′-GCCCCGGAGCCGAGCATTAGCTACAG GAGCGCCGCGGCCG-3′ and reverse 5′-CGGCCGCGGCGCTCCTGTAGCTAATG CTCGGCTCCGGGGC-3′.

Plasmids are available upon request.

### Southern blotting

Bacteria containing the BAC (CH261-43k16) were cultured in 200 ml of 2YT medium for 16 h and DNA was extracted with a Genomed Biotech Kit (Jetstar plasmid purification maxi kit 220020). DNA was digested with PvuII and NcoI for 6 h. DNA transfer was carried out into a nylon membrane (Millipore, immobilon-P membrane PVDF, IPVH00010) and hybridization was performed with two different probes, one to detect the region upstream of the TSS of *Pax2* promoter (Nw_001478025.1) and another one in the 5′UTR of *Pax2* mRNA. The fragment was amplified with specific primers (see the ‘Gene cloning and mutagenesis’ section for the sequence), and the PCR product was purified and labelled with α-P^32^-dCTP (Perkin, NEG5134 250uC) following the manufacturer's instructions (radioactive nucleic acid labelling and detection; GE Healthcare, RPN1633). Hybridization was performed at 65°C overnight and the membrane washed and air-dried for 3 min before exposure to identify the corresponding band that was double positive for both probes.

### ChIP

Fifteen embryos at HH11 were treated following the manufacturer's recommendations (Magna ChIP A; Millipore, 17-610) with 37% formaldehyde solution (Sigma-Aldrich, F1635) and glycine (Merck, 5.00190.1000) with 0.5 ml of cytoplasmic lysis buffer and 0.3 ml of nuclei lysis buffer, both from the Magna ChIP A kit. The antibodies used were IgG as negative control (2 µl, Diagenode, kch-819-015), anti-Histone3 as positive control (2 µg, Ab1791), anti-Pax2 (4 µg; Abcam, Ab23799) and anti-Myc (2 µg; Abcam, Ab9132). For Pax2 interaction with the *Snail1* promoter, embryos were used without any treatment. For Snail1 interaction with the *Pax2* promoter, embryos were electroporated prior to ChIP with 0.5 µg/µl of a Myc-tagged Snail1 plasmid. The primers used for the amplification of the *Snail1* promoter were as follows: forward 5′-CTCCTCGCCCCCCTGTA-3′, reverse 5′-GTACTCGCCCAGCGCCACC-3′. For the negative control NCR, the following primers were used: forward 5′-GCAGCAGCGGCATTATCC-3′, reverse 5′-GTCATGAACCCTTTGGCTTTACC-3′. For amplification of the *Pax2* promoter, the following primers were used: forward 5′-GCTACTCCAGCGCCAACTTA-3′, reverse 5′-GGCCGCGGCGCTCCTGCA-3′.

### Immunohistochemistry

Embryos were washed in 0.1% Triton X-100 in PBS and dehydrated in 25%, 50%, 75% and twice in 100% ethanol for 5 min at RT, then twice in 100% butanol for 15 min and six times in wax for 30 min at 65°C. Sections were cut at 8 µm and hydrated. A permeabilization step was performed with 0.25% Triton X-100 in PBS twice for 20 min each. A blocking solution containing 0.1% Triton X-100 in PBS and 10% fetal calf serum serum was added for 1 h at RT. Anti-GFP antibody (Santa Cruz Biotechnology, SC9996) was added to the blocking solution at 1/1000 and sections were incubated overnight at 4°C. After washing four times for 20 min each wash at RT with 0.1% Triton X-100 in PBS, a secondary antibody (anti-rabbit Alexa Fluor 488 conjugated; Molecular Probes, A-11008) was added at 1/1000 and sections were incubated for 2 h at RT. After washing four times for 20 min each wash at RT, the slides were mounted with Mowiol^®^ 4-88 (Calbiochem, 475904) and imaged using a Leica SPEII confocal with a 10x objective.

## Supplementary Material

10.1242/develop.204848_sup1 Supplementary information
