# Supplementary material for: Mutual repression between Pax2 and Snail factors regulates the epithelial/mesenchymal state during intermediate mesoderm differentiation
Source: Development. 2025 Aug 15;152(16):dev204848. doi: 10.1242/dev.204848 (PMC12401534; doi:10.1242/dev.204848)
Supplement: Supplementary information [file develop-152-204848-s1.pdf]

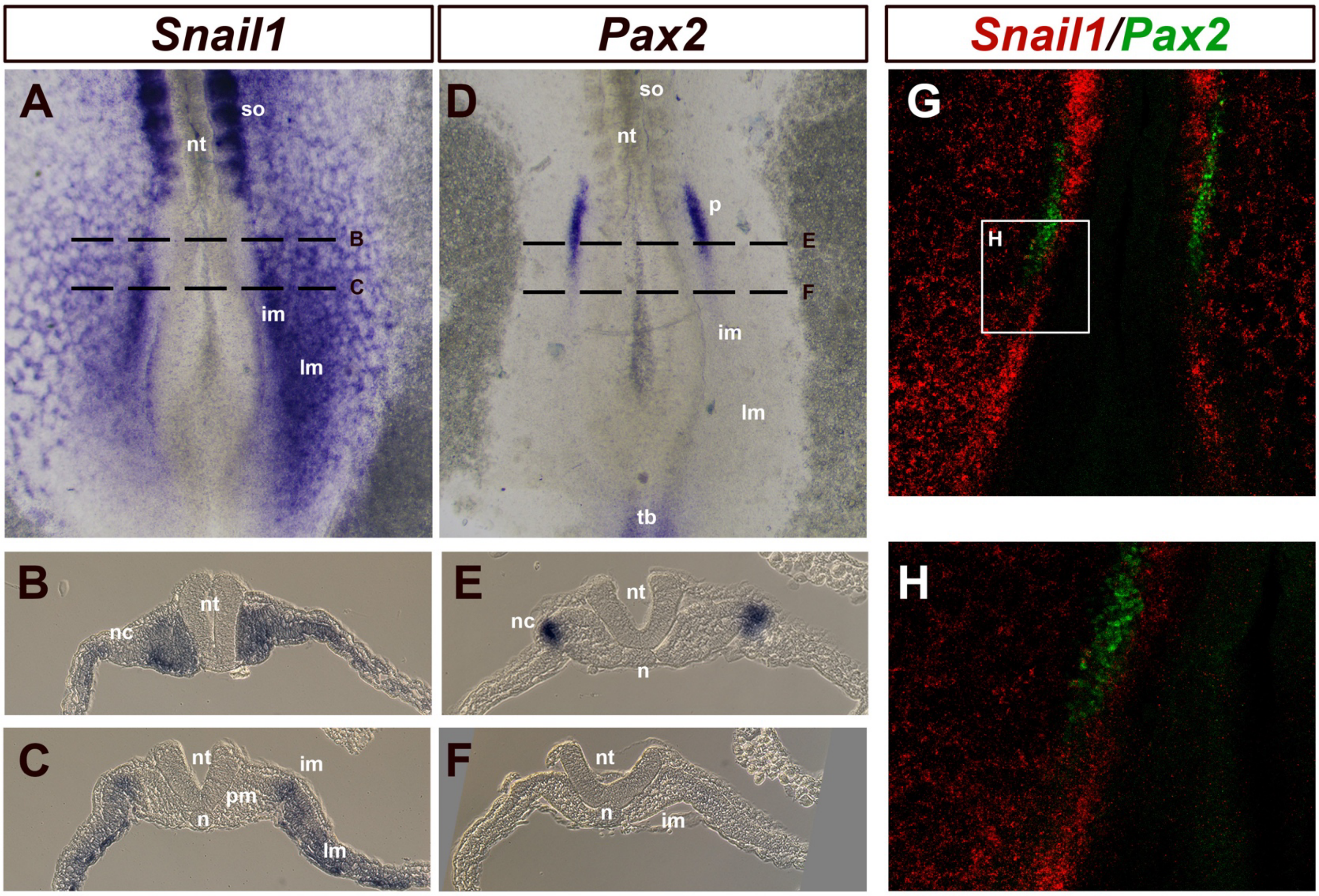

Fig. S1. Snail and Pax2 are expressed in a mutually exclusive pattern in the intermediate mesoderm prior to pronephros formation. (A-F) Dorsal view of chicken embryos at HH9 (8-somite stage) and their transversal sections (B, E, taken at 50 µm posterior to somite +1; C, F taken 100µm posterior to the pronephros front). (A-C) *Snail1* is expressed in the undifferentiated intermediate mesoderm (im), lateral plate mesoderm (lm) and somites (so) (n=10). (D-F) *Pax2* is expressed in the pronephros (p) and in the tail bud (tb) (n=10). (G) 3D reconstruction of a fluorescent *in situ* hybridization for *Snail1* (red) and immunofluorescence for Pax2 (green) (n=3). (H) Higher magnification of the box in G. Note the mutually exclusive expression pattern. n: notochord, nc: nephric cord, nt: neural tube, pm: paraxial mesoderm.

Development • Supplementary information

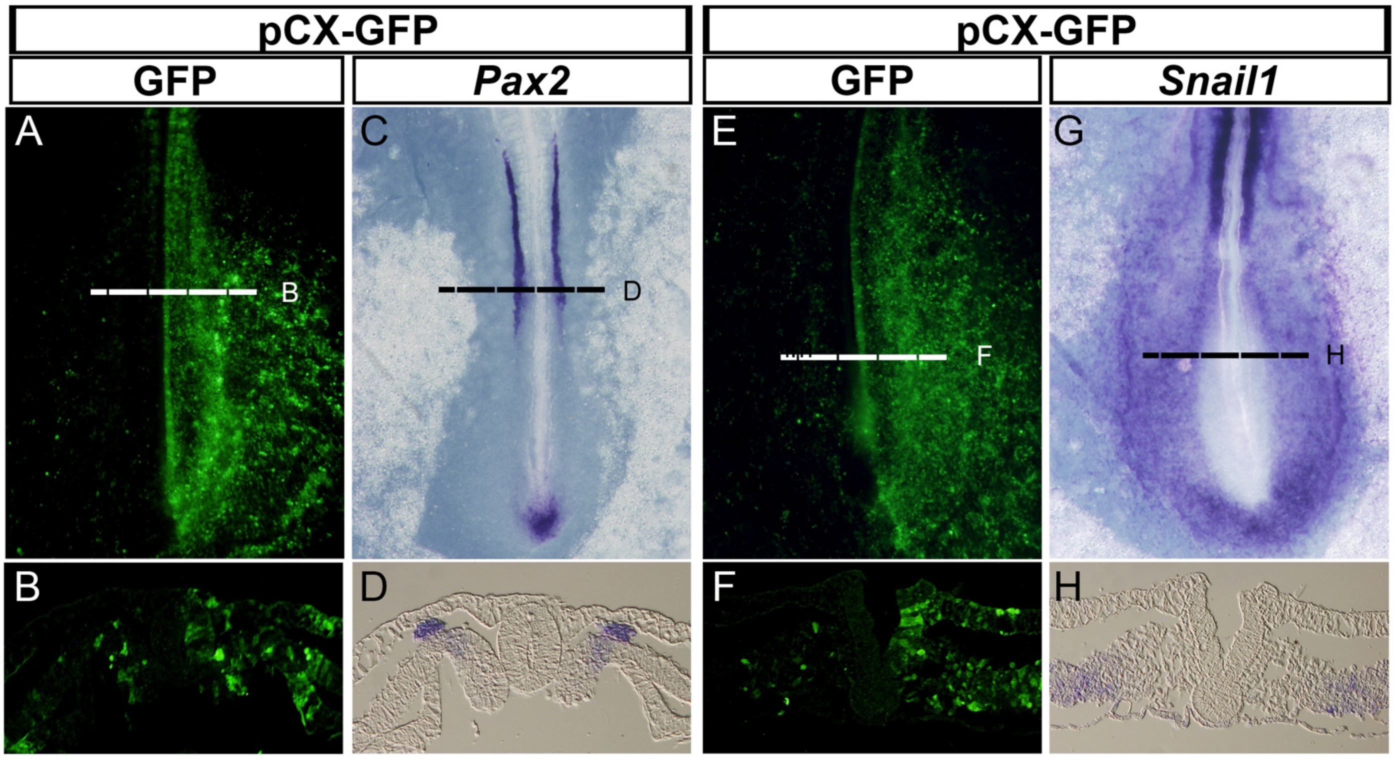

Fig. S2. electroporation controls. Dorsal views of HH11 stage chick embryos and their respective sections (B, D taken at the level of somite +2. F, H taken 500μm posterior to the latest somite formed (+1)). Embryos were electroporated in the right side with pCX-*GFP* and subjected to an ISH for *Pax2* (A-D, n=10) and *Snail1* (E-H, n=10). GFP expression indicates the electroporated regions. There is no change in *Pax2* or *Snail1* expression after the electroporation.

Development • Supplementary information

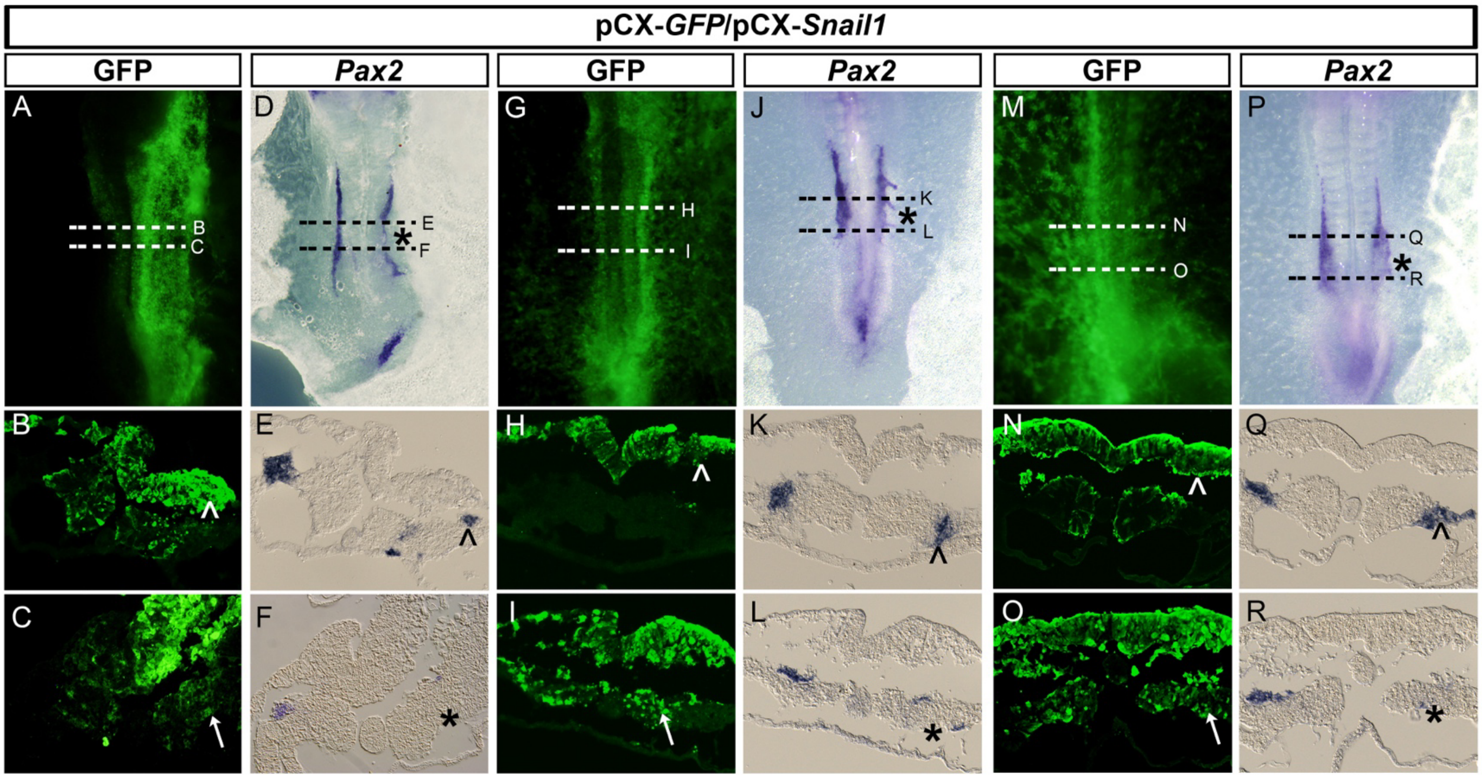

Fig. S3. Snail1 represses *Pax2* transcription. (A-R) Dorsal views of HH11 stage chick embryos and their respective sections co-electroporated in the right-hand side with two vectors, encoding GFP and Snail1. Three representative embryos are shown (A-F, G-L and M-R). (A-C, G-I and M-O) GFP fluorescence. (D-F, J-L and P-R) ISH for *Pax2*. When the IM is not electroporated as seen by GFP (B, H, N, white arrowheads), *Pax2* expression is not affected (E, K, Q black arrowheads). Sections have been taken at somite +3. When it is electroporated (C, I, O white arrows), *Pax2* expression is downregulated (F, L, R, asterisks). Sections were taken at the level of somite -1).

Development • Supplementary information

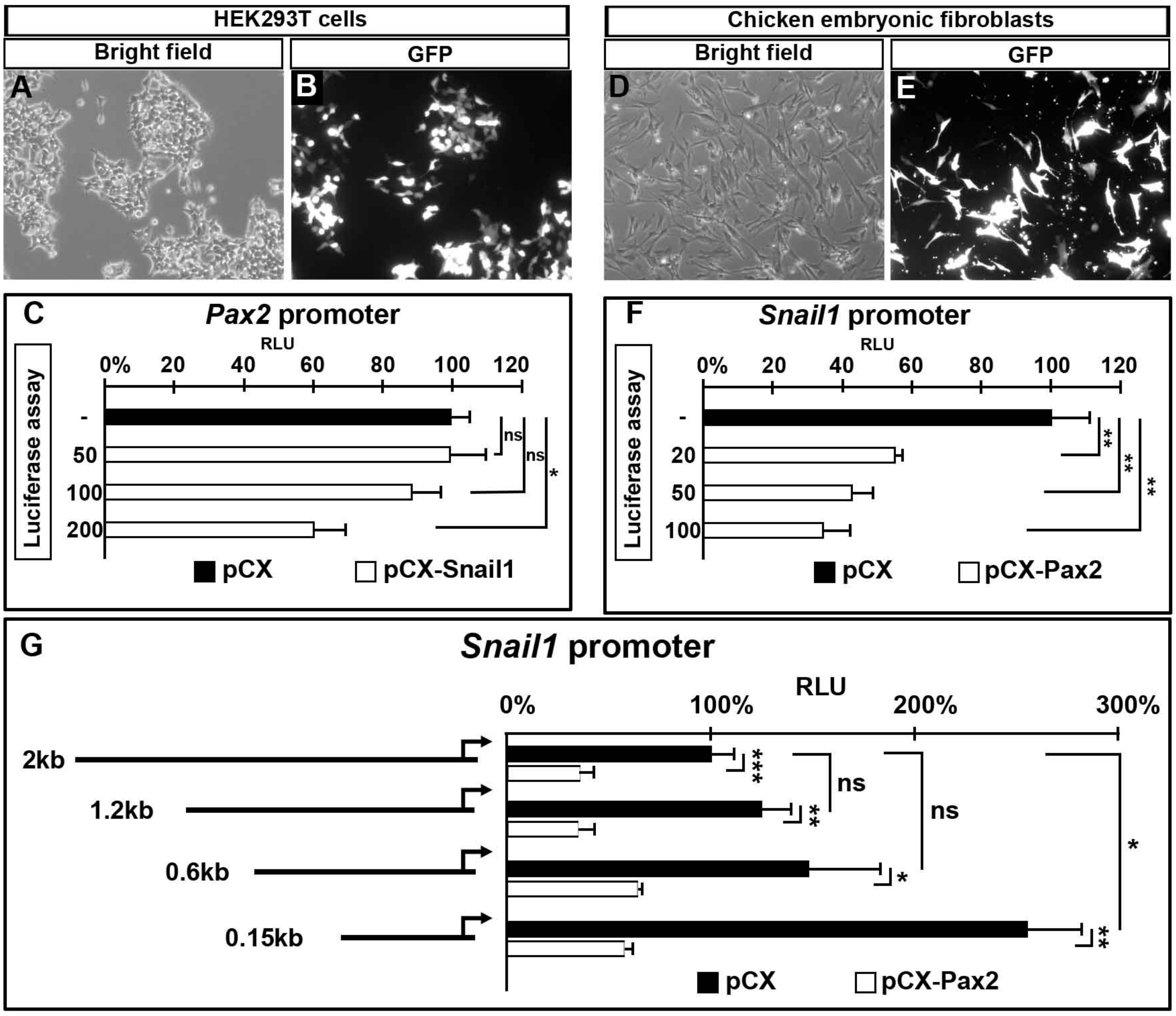

Fig. S4. *Snail1* and *Pax2* promoter analysis. Transfection control with a GFP coding plasmid (pCX-GFP) in HEK293T cells (A,B) or in a primary culture of chick embryonic fibroblasts (CEF) (D,E). (C) Luciferase assay in HEK293T cells after transfecting a plasmid containing a fragment of 1.8kb upstream of TSS of the *Pax2* gene together with increasing concentrations (total ng) of a plasmid coding for Snail1 (pCX-*Snail1,* white bars) or an empty vector (pCX, black bar). The relative luciferase units (RLU) decrease as Snail1 concentration increases. (F) Similar luciferase assay in CEF cells transfected with the corresponding plasmid including sequences of *Snail1* promoter (2kb) and another coding for Pax2 (pCX-*Pax2,* white bars) or pCX control (black bar). Pax2 decreases *Snail1* promoter activity. (G) Deletion analysis of the *Snail1* promoter region. Luciferase assay in CEF cells transfected with plasmids containing fragments of different sizes starting from the TSS (2kb, 1.2kb, 0.6kb and 0.15kb upstream of TSS) and 200ng of pCX-Pax2 vector. Statistics for all experiments: n=3, average representation, T-Test, 2 tails, unequal variance. * p-value ≤ 0.01, **pValue≤ 0.001, ***pValue≤ 0.0001.

Development • Supplementary information

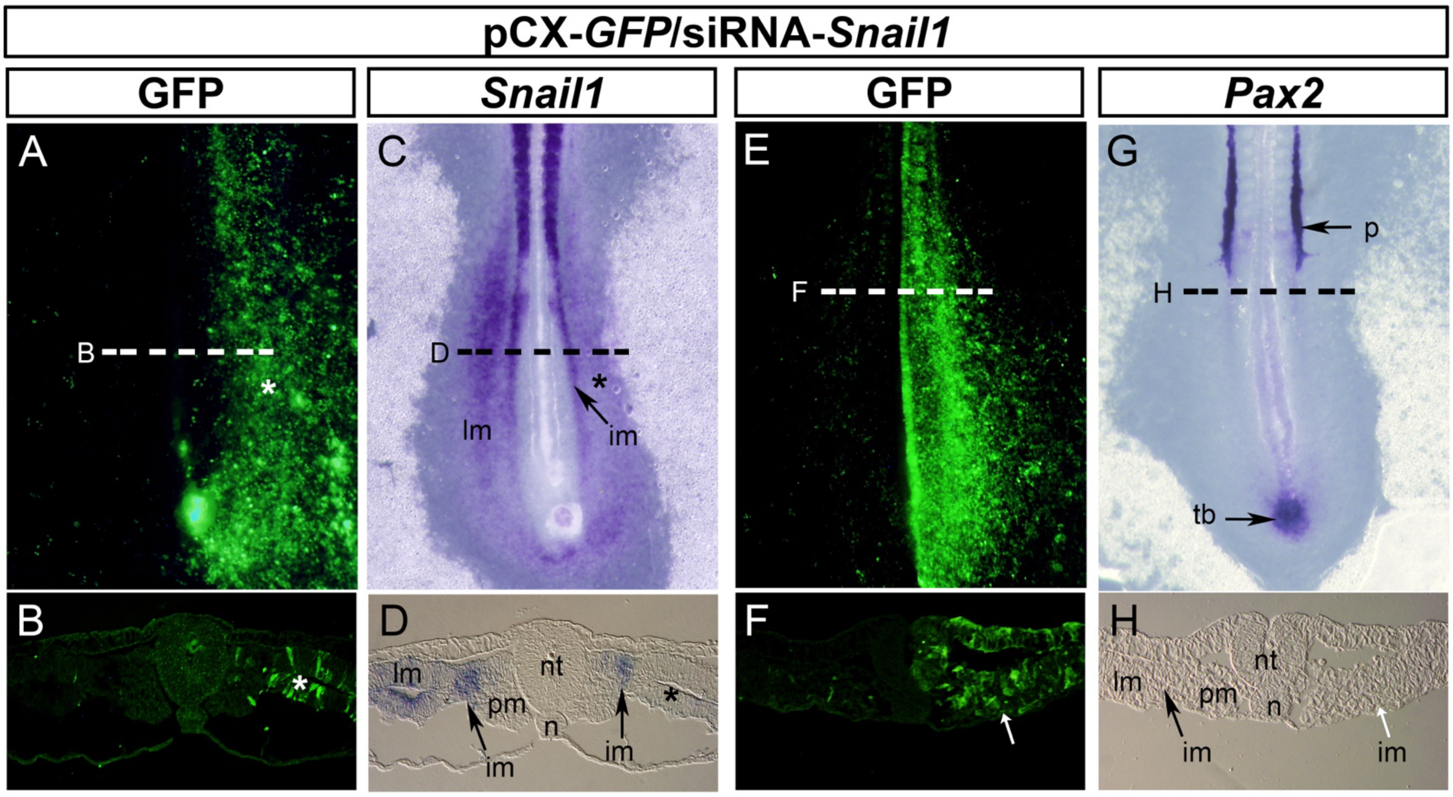

Fig. S5. ***Snail1*** **loss of function is not sufficient to induce** ***Pax2*** **expression.** (A-D) Electroporation of an interference RNA for *Snail1* plus a vector encoding GFP on the right side of chick embryos. (A, B) GFP fluorescence. (C, D) *Snail1* expression. The regions electroporated with siRNA (A, B white asterisk) show downregulation of *Snail1* transcripts in the intermediate mesoderm (C, D arrow) and in the lateral mesoderm (C, D asterisk), validating the effect of the siRNA (n=5/5). E, F) GFP fluorescence. (G, H) ISH for *Pax2*, which is not upregulated in the posterior intermediate mesoderm (F, H, white arrow) in the presence of an siRNA for *Snail1* (E, F, GFP, white arrow) (n=5/5). Sections were taken 500μm posterior to the latest somite formed (+1). n: notochord, nt: neural tube, im: intermediate mesoderm, lm: lateral mesoderm, pm: paraxial mesoderm.

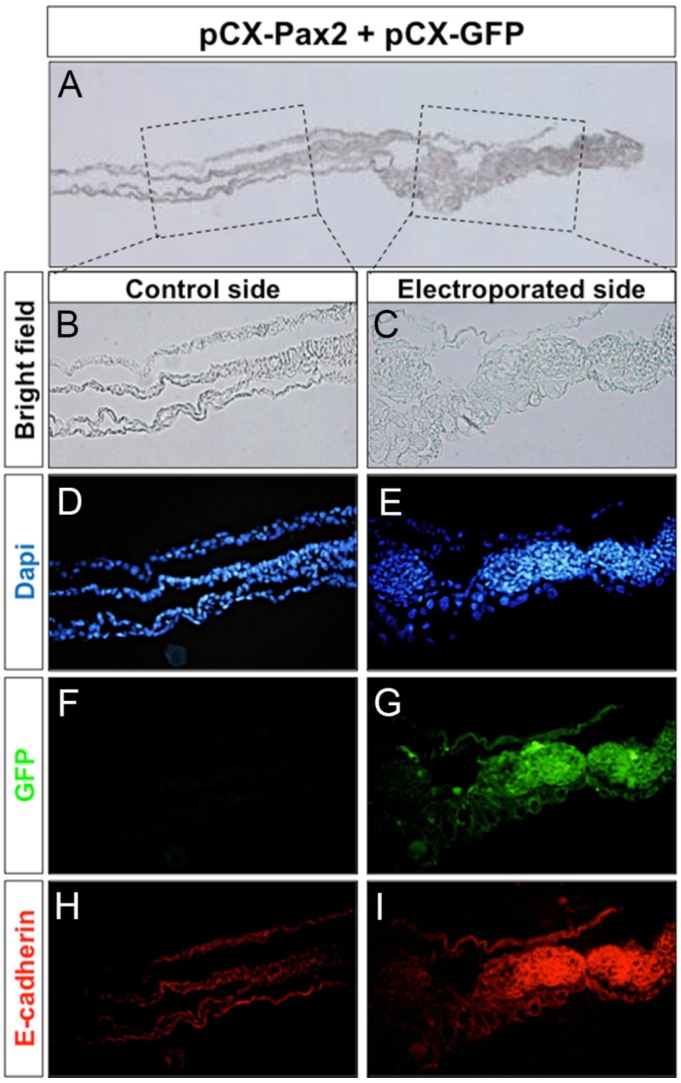

Fig. S6. *Pax2* ectopic expression induces MET in the mesoderm. Electroporation in the right side of a chick embryo with pCX-*Pax2* plasmid plus pCX-*GFP*. (A-C) bright field images taken at a posterior region (500μm posterior to last somite (+1)) of a HH11 stage embryo. The left column corresponds to the non-electroporated control side. (D,E) DAPI staining to show the cell nuclei. (F,G) expression representing the regions of *Pax2* ectopic expression. (H,I) IF for E-Cadherin. Pax2 expression induces the formation of E-cadherin positive epithelial clusters (n=3/3).

Development • Supplementary information

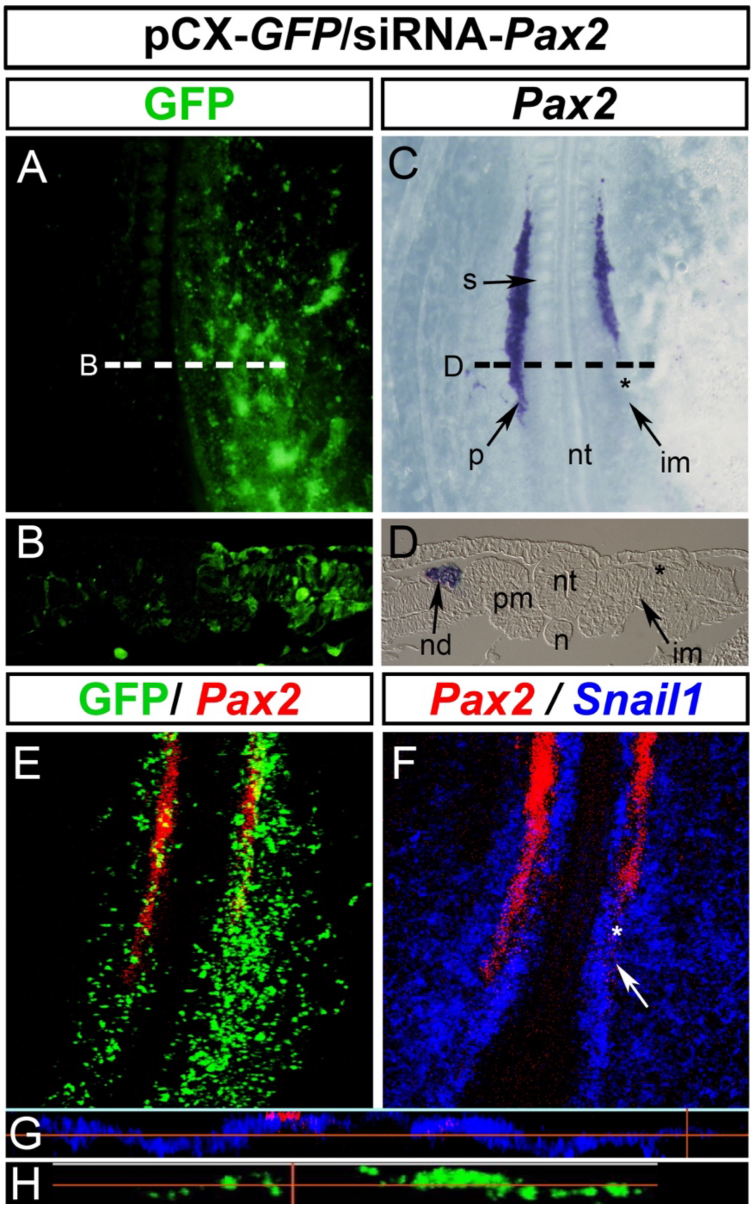

Fig. S7. Pax2 is essential for *Snail1* repression in the intermediate mesoderm during pronephros differentiation. Electroporation of RNA interference for *Pax2* plus a vector encoding GFP on the right side of chick embryos shown at HH11. A, B) GFP fluorescence. C, D) ISH. *Pax2* expression (section taken at the level of somite +1). The regions electroporated with iRNA (A, B, GFP) show downregulation of *Pax2* transcripts (C,D, asterisk), validating the effect of the siRNA (n=5/5). (E) GFP fluorescence (green) and ISH for *Pax2* in red. (F) Double fluorescent ISH for *Pax2* in red and *Snail1* in blue (same embryo as in E). The presence of siRNA for *Pax2* (E, green) impaired *Pax2* expression (white asterisk) while *Snail1* expression was maintained in the intermediate mesoderm (compare the white arrow with the white arrowhead in the contralateral side; n=5/5). G and H) Optical transversal sections (taken at somite +1) showing that Pax2 downregulation leads to *Snail1* maintenance in the electroporated side. See single positive Pax2 expressing cells in the the contralateral side n: notochord, nd: nephric duct, nt: neural tube, im: intermediate mesoderm, pm: paraxial mesoderm.

Development • Supplementary information

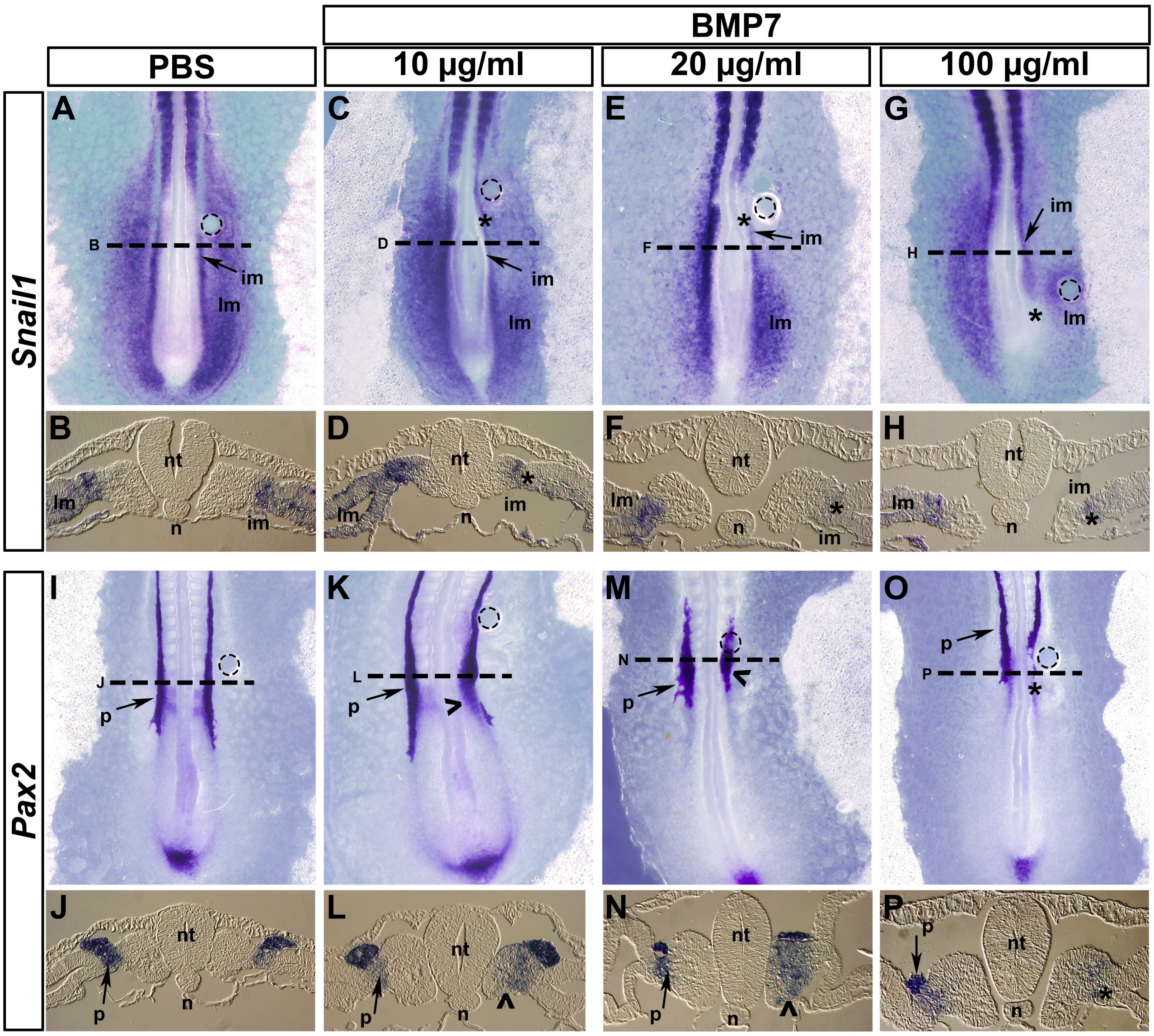

Fig. S8. BMP7 represses *Snail1* and induces *Pax2* transcription in the bi-stability domain. Dorsal view of HH11 stage chick embryos and their respective sections subjected to ISH for *Snail1* (A-H, all sections taken 500µm posterior to the latest somite formed (+1)) or *Pax2* (I-P, all sections taken at somite +2). (A-P) PBS or BMP7-soaked acrylic beads were implanted in control embryos (A,B,I,J n=5) or experimental embryos (C-H and K-P), respectively (dotted circles). (A-H) BMP7 represses *Snail1* transcription in the intermediate (im, asterisks) and lateral mesoderm (lm) at all concentrations (C, n=8/16; E, n=5/20; G, n=4/8). At high concentration (100 µg/ml) *Snail1* is not repressed in cells close to the bead. This is likely due to Bmp7 acting as Bmp2 or Bmp4, known to induce *Snail1* expression. In more distant regions, where lower concentrations should be available, *Snail1* expression is still repressed. (I-P) BMP7 induces *Pax2* transcription within the bi-stability domain in the presomitic mesoderm (arrowhead) (K, n=10/17; M, n=12/35; O, n=13/19). At the highest concentration, BMP7 inhibits *Pax2* expression in the intermediate mesoderm (O,P, asterisks, n=13/19), likely due to the observed upregulation of *Snail1* expression at this high concentration and to the known role of a BMP gradient in specifying different mesodermal populations along the mediolateral axis. n: notochord, nt: neural tube, p: pronephros.

Development • Supplementary information

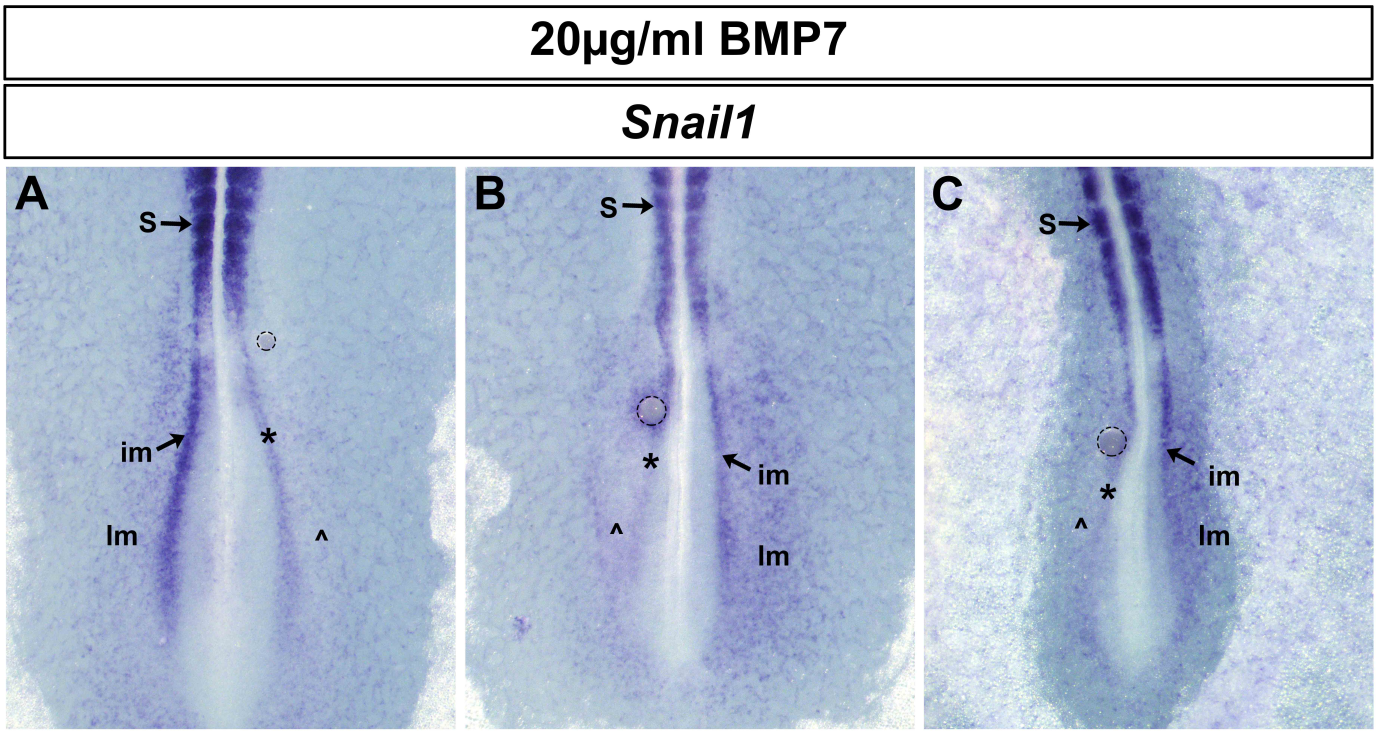

Fig. S9. BMP7 represses *Snail1* expression. Dorsal views of HH11 stage chick embryos with a BMP7 soaked bead (dotted circle), either in the right (A) or the left side (B,C). *Snail1* is downregulated in the intermediate (asterisk) and lateral mesoderm (arrowhead). im: intermediate mesoderm, lm: lateral mesoderm, s: somite.

Development • Supplementary information

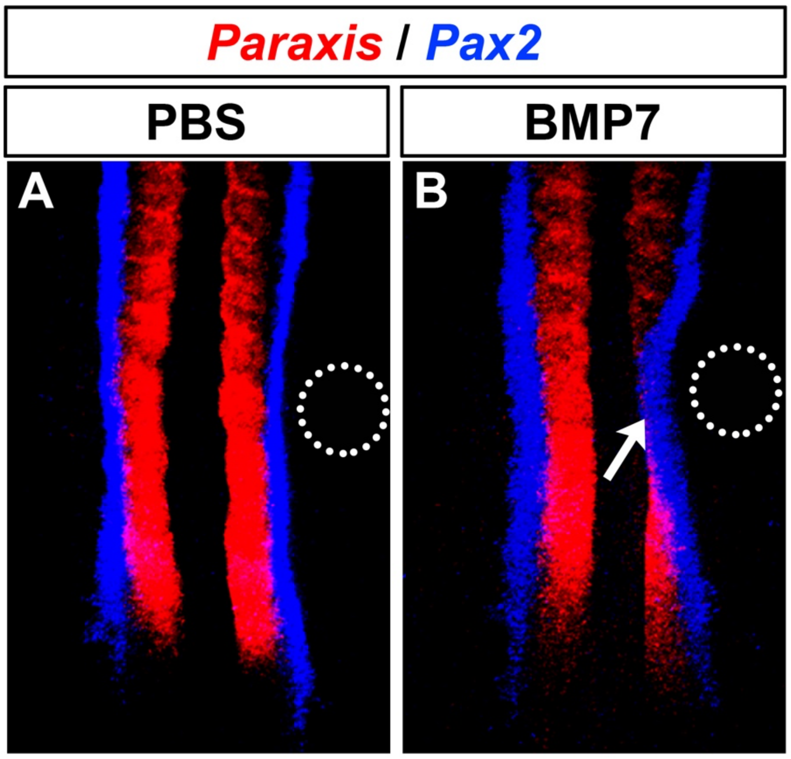

Fig. S10. BMP7 induces intermediate mesoderm fate in the paraxial mesoderm. 3D Reconstruction of a double fluorescent *in situ* hybridization for *Pax2* (blue) and *Paraxis* (red) in a HH11 stage chick embryo. The addition of 20 ng/ml of BMP7 (B, dotted circle) alters the mediolateral patterning of the embryo (arrow) as seen by *Paraxis* downregulation and *Pax2* upregulation in the same territory (n=5/6).

Development • Supplementary information

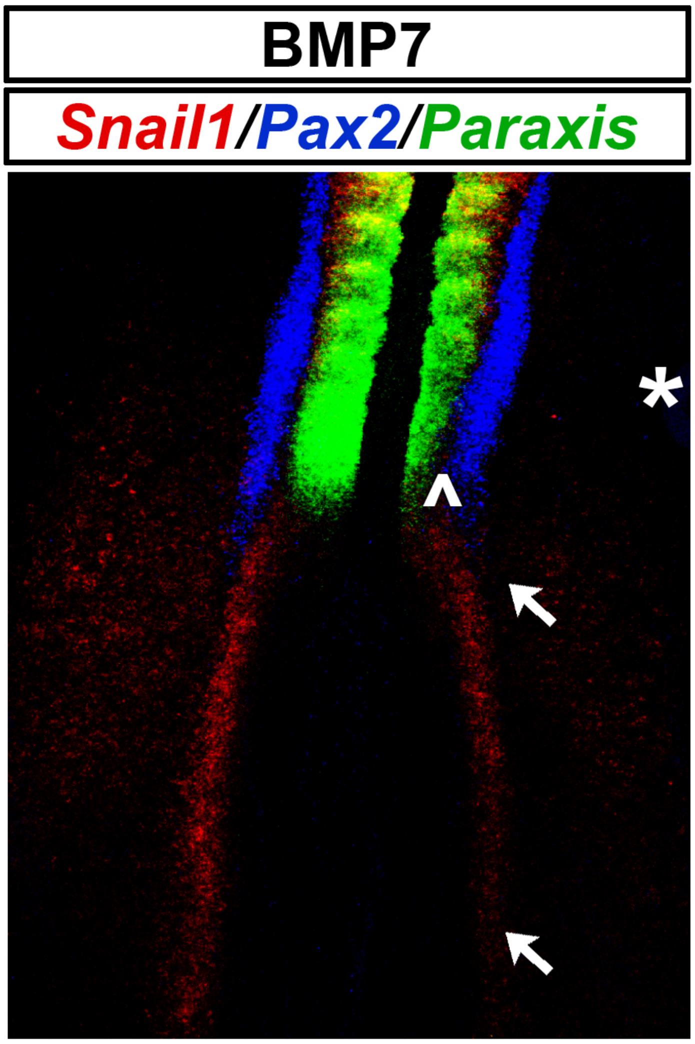

Fig. S11. BMP7 represses *Snail1* independent of *Pax2* induction. 3D reconstruction of a HH11 stage chick embryo with a triple fluorescent ISH for *Snail1* in red, *Pax2* in blue and *Paraxis* in green. A BMP7-soaked bead was implanted on the right side (asterisk). BMP7 represses *Snail1* in territories that are *Pax2* negative (arrows). *Pax2* is induced in the paraxial mesoderm where *Paraxis* is downregulated (arrowhead).

Development • Supplementary information

Supplementary Materials and Methods

Pax2 promoter cloned sequence
Black is the UTR, red is the probe sequence target, green is new sequence and blue is the Nw_001478025.1 contig.

5'TCT CCG AGA TTC TTT ACT TAA AGC AGG CAG ACA ACA AGG GAC CGT TCC GTG GCA GAT AGC GGAGGG GTG GGG AAG GAG GAA AAG AG ACG GAG AAG TGT ATT TCG ACG GGT GAT CGG CCC GCA GTC TGC CGA ACA AAA TTC AGC TCC CAG CAG TTT CTG CCT CGT CTT CCC ACC TCT GGT CCT CAG CCT CAC AGG CGC TCC TTC CCC TCT CTC TGT TCC TTG CTA GGG CGC AGC TAC GTC TAT TTT AGC CGT CTA TCA GCT TCT GAA ATG ATT TTT AAA TCC CAC TTT AGC TCA ACA TTA CGA AAA AAA AAA AAG AAA GAA ACC AAA TGA AAA ATA AAA AAG ATG TGG AAA GTG GGA AGT TGA GTT CCT TTG TTT TTC TCA AAC GTG CAA GTG AAA CGT GCA AGT GGA AAC TTT GCC ATA ATT GAA ATG ATC TAT TGG TAC AAA GAG ATA GAA GGT CTT GCC TCC CTT CCT TCA CTA TTT TTC TTC TTT CTT TAA TTT ATT TCA ATT TTC TTT CTG TTA TCC TTT CTT TCT TTC TTT CTT TCT TTC TTT CTT TCT TTC TTT CTT TCT TTC TTT CTT TCT TTC TTT CTT TCT TTC TTT CTT TCT TTC TTT CTT TCT TTC TTT CTT TCT TTC TTT CTT TCC TCT CTC CTCT TCC TTC CTT CCT TCC TTC CTT CCT CTC TTT CCT TCC TCT CTT TCC TTC TTC TCT TTC CTT TTT CTC TTT CTC TCT TCCTCT CCG TCT TTC ACC TTT CCT ATT CCT CTC CTT CTC TCT TTC TCC TTT CCT CTT CCC TTC TTT ATT CAC TTC TTT CTC CCT TTC TCT TCC TCT CCG CTA CCC TTT CCC TCC GTT CCC GCT GGC CCC GAT GCG TAC GGA CAC CCC CTC CCC GCT CCA GCC CGA GCG AAG TCT CCA GCA GCA GCG AGG AGA TGG AAG CAC CGA GCG GGG CCG TGC GAG CAG CGG CAG CCC CGG CAC GGA TCG CGG CGA GCC CGG CGC AGG CCG AGC TCT GTG AGG CGC TGG GGC CGG GCC GGG CCG GGC CGC CCT CCC TGC GCC CTC CGG GGC CCC GCG GCG ACC GGG CCG CCC CCG CAC CGC GTC CCG GCG GCG GCT GCG GCC CCC CCG CGC CGA GCG GCG GCC CGG CCC TCC TCG CCG CGC TCG GCT CGG CGC GCT GGC GAA TCA GAG AGT GTC GGA ATC TAT TGC CTTTGT CTG ACA AGT CAT CCA TCT CCG GGG AGG CGG GCG GGG GGC TGA GGG CGC TGC GGC TTT TAG AGA GAC ACA CAC CGG GAG AGG AGG CTC CAG TCT CCG GCC CCG GCT CCT CGC AAG CAC TTC CCA CTC GGG CGC AAG TCC GCC GGG CGC CGG AGC TTC CGC CCG GCC GAG AGG AGG GGC GCG CCC GGC CGC CGC TCG CCG CTT TCC CGG GGA TTG CTA CTC CAG CGC CAA CTT ACG AAC TGC CCC GGA GCC GAG CAG CAG CTG CAG GAG CGC CGC GGC CGC GCC GCC CGC CCC CCT CCC CGC CGC CGG GCC CCG GGC CCC AGC CAT GCC CGC CGC CTG CCC CCC GGC GCC GCC AGC CCC GAG CCC CCG CCG CCC CCG CTG AGC GCC CGC CCG GGA AGC GCC GGC CCC GCG CCC CGG CCC GGC CGG CGC CGC TCC TGC GCT CCT CTC CCT CTG TCT GCT TTT CGC TTT GGT TTT ATT TTT GT TTG CTT TCG ATT GCT TTG CTT TGG TTT GTT ATT TTG GGG TTT TGG GTT TTT TTT CGT TTT TTT TTT TGT TTG TTT GTT TGC TTA TTT TTT TCT CCC CTA AGT CTT GAA GTT GAG TTT TAG AGG CGA CAC GGC GGC TTC AGC CGA TTT CTT CCC TTT CCT TTG CCT CCC C ATG 3'
